# The mitochondrial genome of *Calohilara tibetensis* Ding, He, Lin, and Yang, 2020 (Diptera: Empididae)

**DOI:** 10.1080/23802359.2021.1962212

**Published:** 2021-08-11

**Authors:** Chen Lin, Shang Gao, Ding Yang

**Affiliations:** a Institute of Life Science and Technology, Inner Mongolia Normal University, Huhhot, PR China; b College of Plant Protection, China Agricultural University, Beijing, PR China

**Keywords:** Mitochondrial genome, Empididae, *Calohilara tibetensis*, phylogenetics

## Abstract

The dance fly *Calohilara tibetensis* Ding, He, Lin and Yang, 2020 belongs to the subfamily Empidinae of Empididae. The mitochondrial genome of *C. tibetensis* was sequenced as the new representative of the subfamily Empidinae. The nearly complete mitogenome was 15,354 bp, consisting of 13 protein-coding genes, two *rRNA*s, and 22 *tRNAs*. All genes have the similar locations and strands with that of other published species of Empididae. The nucleotide composition biases toward A and T is 77.4% of the entirety. All PCGs start with ATN codons except *COI* and *NAD1*, and end with TAA or incomplete stop codon. Bayesian inference (BI) analysis strongly supported the monophyly of both Empididae and Dolichopodidae and the monophyly of subfamily Empidinae. It suggested that *Calohilara* is the sister group of *Hilara*.

## Introduction

Empididae is one of the largest families in Diptera, including over 5000 extant species in over 180 genera and subgenera (Yang et al. 2007). They capture aphids, psyllids, and coccids of Hemiptera, but also other true flies, such as agromyzid flies, mosquitos, blackflies, and so on. They are widely used as a biological indicator of evaluating the quality of environment and biodiversity (Yang and Yang 2004).

The specimens of *Calohilara tibetensis* Ding He, Lin and Yang, 2020 (Voucher number: CAU-YDLCEMPI-Calo-1, Liang Wang, 1352659341@qq.com) used for this study were collected from Renqingbeng in Motuo of Tibet (29°33′N, 95°33′E) by Qicheng Yang on 1 Jun 2019, and then identified by Shuangmei Ding through the morphological characters (thoracic pleuron blackish except sternopleuron with a brownish postero-dorsal spot; wing brown to dark brown with one pale spot at middle; fore and mid femora with thin pv hair-like; hind femur with thin av hair-like; hypandrial processes rod-like, apically trifurcated) (Ding et al. 2020). Specimens were preserved in 100% absolute ethanol and stored at −20 °C refrigerator in the Entomological Museum of China Agricultural University.

The total genomic DNA was extracted from adult’s whole body (except head and wings) using the DNeasy DNA Extraction kit (TIANGEN Biotech Co. Ltd, Beijing, China). The DNA samples extracted from four conspecific individuals were pooled for next-generation sequencing library construction following the method of Gillett et al. (2014). All quantified DNA extracts were included in a single pool at equimolar concentration, aiming for 50 ng/µl of dsDNA per sample, resulting in a DNA pool of approximately 5 µg. The library was sequenced on an Illumina HiSeq 2500 by BIONONA CO., LTD. Raw read data were trimmed and cropped in Trimmomatic version 0.30 with the default setting (Bolger et al. 2014). 4GB of high-quality reads were used to assemble mitochondrial genome with the *de novo* assembler IDBA-UD (Peng et al. 2012). The bait sequence *COI* was amplified by standard PCR reactions. BLAST search was carried out with BioEdit version 7.0.5.3., and the position of all *tRNA* genes was confirmed using tRNAscanSE version 2.0 (Lowe and Chan 2016).

The nearly complete mitochondrial genome of *C. tibetensis* is 15,354 bp in length (GenBank accession number: MW438868). It contains 13 typical protein-coding genes, 22 *tRNA* genes, and two *rRNA* genes (12S *rRNA* and 16S *rRNA*), but the control region could not be sequenced entirely in this study, which were similar with related reports before (Wang et al. 2016; Gao et al. 2018; Hou et al. 2019; Liu et al. 2020). All genes have the similar locations and strands with that of other published Empididae species. The nucleotide composition of the mitochondrial genome was biased toward A and T, with 77.4% of A + T content (A = 39.6%, T = 37.8%, C = 13.7%, G = 8.9%). Among the protein-coding genes, six genes took the start codon of ATG (*COII*, *COIII*, *ATP6*, *NAD4*, *NAD4L*, and *CYTB*), five genes used ATT (*NAD2*, *ATP8*, *NAD3*, *NAD5*, and *NAD6*) as start codon, while *COI* gene and *NAD1* gene got TCG and TTG, respectively. Only *NAD5* used T + tRNA as a termination codon, the other protein-coding genes ended with the canonical TAA codon. The length of *tRNA* genes ranges from 63 to 70 bp. All *tRNA* genes can be folded into the typical clover-leaf secondary structure. The 16S rRNA is 1357 bp in length with A + T content of 82.7%, and the 12S rRNA is 795 bp long with A + T content of 78.6%.

**Figure 1.**
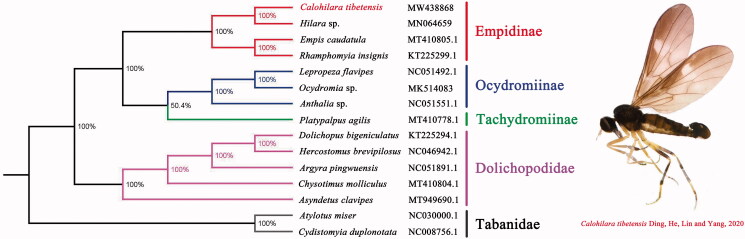
Bayesian phylogenetic tree based on 13PCGs of 15 Diptera species including *Calohilara tibetensis*. Genbank accession numbers of all sequence used in the phylogenetic tree have been included in the figure and corresponding to the names of all species. Red words indicated new sequenced data in this study.

The phylogenetic analyses were performed using Bayesian inference (BI) under GTR model in MrBayes version 3.2.7a (Ronquist et al. 2012) based on the concatenated dataset (PCGs) of mitogenomes of *C. tibetensis* and other 14 taxa (Figure 1). The result showed that the monophyly of Empididae and Dolichopodidae were strongly supported, which was consistent with the phylogenetic result of the previous research (Wang et al. 2016). The monophyletic Empididae that contains Empidinae, Ocydromiinae, and Tachydromiinae was assigned as the sister group to the clade of Dolichopodidae in this study. For the phylogeny within Empidinae, it suggested that *Calohilara* is the sister group to *Hilara*. The mitogenome of *C. tibetensis* may provide essential and important DNA molecular data for further phylogenetic and evolutionary studies of subfamily Empidinae and family Empididae.

## Data Availability

Mitogenome data supporting this study are openly available in GenBank at https://www.ncbi.nlm.nih.gov/nuccore/MW438868, Associated BioProject, https://www.ncbi.nlm.nih.gov/bioproject/PRJNA717633, BioSample accession number at https://www.ncbi.nlm.nih.gov/biosample/SAMN18499429, Sequence Read Archive at https://www.ncbi.nlm.nih.gov/sra/SRR13908658.
